# Long-Term Effect of Enzyme Replacement Therapy with Fabry Disease

**DOI:** 10.1155/2013/282487

**Published:** 2013-10-08

**Authors:** Manabu Komori, Yuika Sakurai, Hiromi Kojima, Toya Ohashi, Hiroshi Moriyama

**Affiliations:** ^1^Department of Otorhinolarygology, The Jikei University School of Medicine, 3-25-8 Nishishinbashi Minato-ku, Tokyo 105-8461, Japan; ^2^Department of Gene Therapy, Institute of DNA Medicine, The Jikei University School of Medicine, Tokyo 105-8461, Japan; ^3^Department of Pediatrics, The Jikei University School of Medicine, Tokyo 105-8461, Japan

## Abstract

*Objective.* To determine the effects of enzyme replacement therapy (ERT) on the hearing acuity in patients with Fabry disease. *Materials.* The study sample comprised 34 ears of 17 affected patients who underwent pure-tone audiometry before and after ERT. *Methods.* The patients were studied in relation to factors such as changes in hearing, presence of accompanying symptoms, status of renal and cardiac function, age, and gender. Data of pure-tone audiometry obtained before ERT and at the final examination were compared. *Results.* At the end of the follow-up period, no significant worsening of hearing acuity was noted at the end of the follow-up period. SSNHL was detected in 10 ears of 6 patients. Steroid therapy successfully cured the disease in 9 of the 10 ears. *Conclusions.* No significant worsening of hearing acuity was noted from the beginning to the end of ERT. The rate of improvement in SSNHL of Fabry disease was excellent in the treated patients. Hearing loss is a factor that causes marked deterioration of the patients' quality of life, and it is desirable that the hearing acuity of patients be periodically evaluated and prompt treatment of SSNHL be administered, if available.

## 1. Introduction

Fabry disease is a genetic inborn error of metabolism in which the enzymatic activity of *α*-galactosidase (*α*-Gal), a hydrolytic enzyme present in lysosomes, is decreased due to a gene mutation; this results in the accumulation of glycolipids, mainly in the vascular endothelium. This disorder was first reported in 1898 by 2 independent investigators, namely, Anderson from the UK and Fabry from Germany [1, 2]. The disease is acquired by X chromosome-linked inheritance, and male and female patients with Fabry disease are hemizygous and heterozygous, respectively. Further, male patients with Fabry disease can either present with the classic type of the disease or the late-onset subtype.

The disease mainly involves the kidney, heart, and brain, and accordingly, affected patients often die in their 40 s or 50 s because of renal failure, heart failure, or cerebral infarction. In addition, angiokeratoma and hypohidrosis are present, with a variety of neurologic symptoms, including severe pain in the extremities, burning sensation, headache, dizziness, hearing loss, lack of motivation, and neurosis. According to the published literature, 54.5% [3] to 80% [4] of patients with Fabry disease experience hearing loss. In a previous study, we examined the relationship between hearing acuity and complications occurring in Fabry disease patients treated at our institution and found that 44.4% of the patients had hearing loss [5]. We reported that flat-type hearing loss was predominant in both male and female patients, that all patients with renal dysfunction had hearing impairment, and that the incidence of hearing impairment was higher in men than in women [5].

As agents for enzyme replacement therapy (ERT), *α*-Gal*β* (Fabrazyme) and *α*-Gal*α* (Replagal) were approved in Europe in 2001 and in the USA in 2003. In Japan, Fabrazyme was approved in 2004 and Replagal in 2007. Early initiation of ERT has been reported to prevent or improve renal failure and heart failure [6, 7]. Studies from Europe and North America have reported the beneficial effect of ERT on hearing impairment, as long as it was not very severe [4, 8]. However, the course of changes in hearing remains unclear. With this in view, we sought to determine the changes in hearing affected by ERT in patients who had normal hearing acuity before the initiation of the therapy.

## 2. Materials

 In this study, we examined 34 ears of 17 patients (8 men and 9 women) with Fabry disease, who underwent pure-tone audiometry before and after ERT. All these patients had a normal mean hearing level of less than 20 dB before the initiation of ERT. The mean age of the patients at the first visit was 35.0 years (31.0 years in men, 38.6 years in women), and the mean follow-up period was 46.6 months (8–90 months).

## 3. Methods

 The patients were investigated for the course of changes in hearing and the severity of accompanying symptoms (sudden sensorineural hearing loss (SSNHL), tinnitus, and vertigo); different groups of patients were compared in terms of factors such as renal and cardiac functions, age, and gender. Hearing impairment was evaluated by pure-tone audiometry by using the five-division method, (250 Hz + 500 Hz + 1000 Hz + 2000 Hz + 4000 Hz)/5, according to the diagnostic criteria for sudden hearing loss in Japanese patients. The results of pure-tone audiometry performed before starting ERT and at the final follow-up examination were compared. The severity of SSNHL and the outcome of therapy were classified according to the classification criteria set for assessing sudden hearing loss in Japanese patients (Tables 1 and 2). When the difference between the mean hearing levels recorded before and after ERT exceeded 5 dB, hearing impairment was deemed present. Renal failure was defined by increased levels of serum urea nitrogen (>20 mg/dL), increased levels of serum creatinine level (>1.2 mg/dL), or if the patient received renal dialysis. To evaluate cardiac function, patients were examined for myocardial hypertrophy in terms of the thickness of the myocardium in the interatrial septum and posterior wall, as determined by echocardiography or cardiac MRI. Cardiac damage was diagnosed when the patient had a history of myocardial hypertrophy, cardiac ischemic events, or pacemaker implantation. This study was approved by the Jikei University School of Medicine Ethics Committee.

## 4. Results

### 4.1. Overview

**Figure 1:**
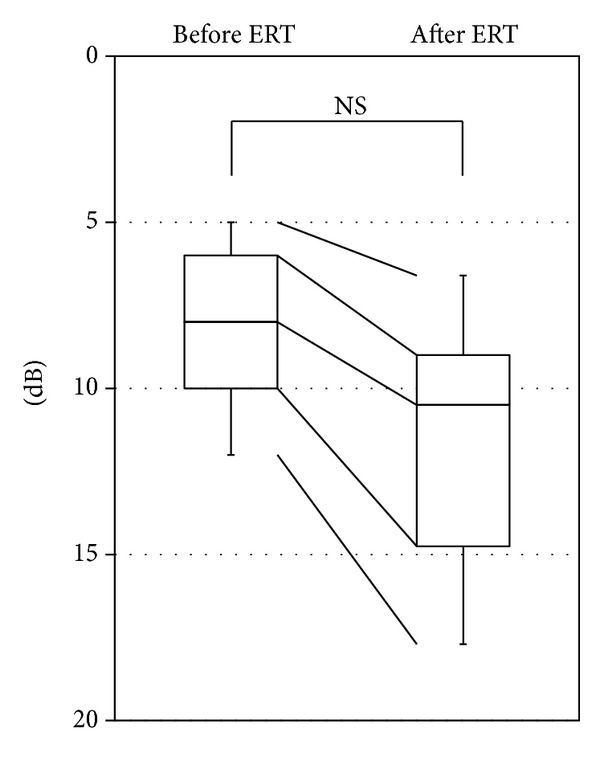
Changes in the hearing level after ERT. None of the patients showed significant worsening of hearing from the beginning of ERT until the end of followup.

Data collected over the study period are presented in Table 3. Hearing impairment was noted in 7 ears of 5 patients (>5 dB) at the end of the follow-up period. The impairment had progressed slowly in the case of 4 (all in men) of the 7 ears, whereas SSNHL occurred in the remaining 3 ears (1 man and 2 women). However, the mean hearing level was less than 30 dB in all these 5 patients, thereby eliminating the need for hearing aids. None of the patients showed significant worsening of hearing (>5 dB) from the beginning of ERT until the end of followup (Figure 1; *χ* ^2^ test). Thirteen of the 17 patients had tinnitus, while 9 (4 men, 5 women) had vertigo. SSNHL was found in 10 ears of 6 patients (3 men, 3 women). The severity of SSNHL was grade 1 in 9 ears and grade 3 in 1 ear. The condition was judged as cured in 9 of the 10 ears after steroid therapy, on the basis of our criteria for improvement. Repetitive SSNHL was found in 3 (1 man and 2 women) of the 6 patients with SSNHL. The incidence of SSNHL tended to increase over the follow-up period, and all patients with renal dysfunction had SSNHL.

### 4.2. Patients with SSNHL

**Figure 2:**
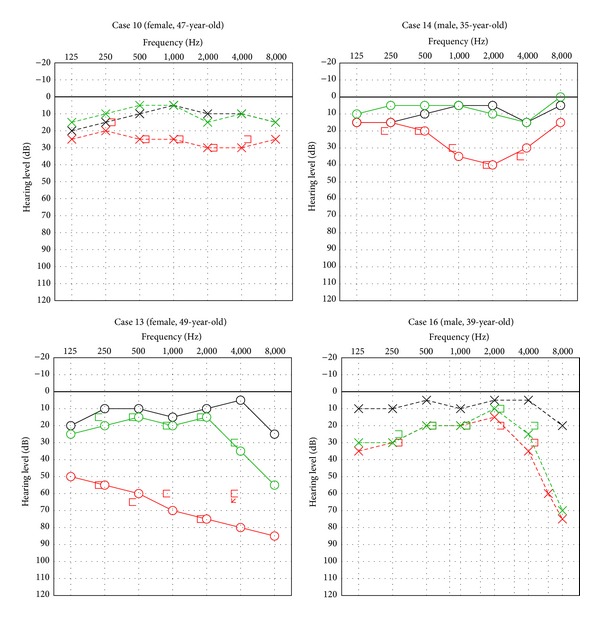
Four cases of sudden sensorineural hearing loss. Black line: at the first visit (before ERT). Red line: at the onset of SSNHL. Green line: after steroid treatment.

The findings of typical cases of SSNHL are presented in Figure 2. Various patterns of hearing loss were noted among the patients, such as whole-area involvement, valley-type hearing loss, and sudden drop in high-frequency hearing.

#### 4.2.1. Case 10 (A 47-Year-Old Woman)

 The patient developed SSNHL (grade 1) on 1 side at 41 months of age and on the opposite side at 54 months of age; at both instances, the patient was cured by steroid therapy. 

#### 4.2.2. Case 14 (A 35-Year-Old Man)

 SSNHL (grade 1) first occurred when the patient was 48 months of age and then again reoccurred at the opposite side at 60 months of age; both ears responded to steroid therapy. This patient showed no symptoms of the Fabry disease other than hearing loss.

#### 4.2.3. Case 13 (A 49-Year-Old Woman)

 In this case, the patient developed SSNHL first at 20 months of age with grade 1 severity and then at 67 months with grade 3 severity. The condition was successfully treated with steroid therapy at both instances.

#### 4.2.4. Case 16 (A 39-Year-Old Man)

SSNHL (grade 1) occurred at 84 months of age and did not respond to steroid therapy.

## 5. Discussion

Since the Fabry disease is rare and shows diverse symptoms in the initial stages of manifestation, the definitive diagnosis of this disease was often delayed in the past. However, in recent years, Fabry disease has become more widely recognized, and therefore the disease is now increasingly detected at an early stage and ERT is soon initiated. In this study, all the enrolled patients had normal hearing of less than 20 dB before ERT, and the mean follow-up period was approximately 4 years. 

**Figure 3:**
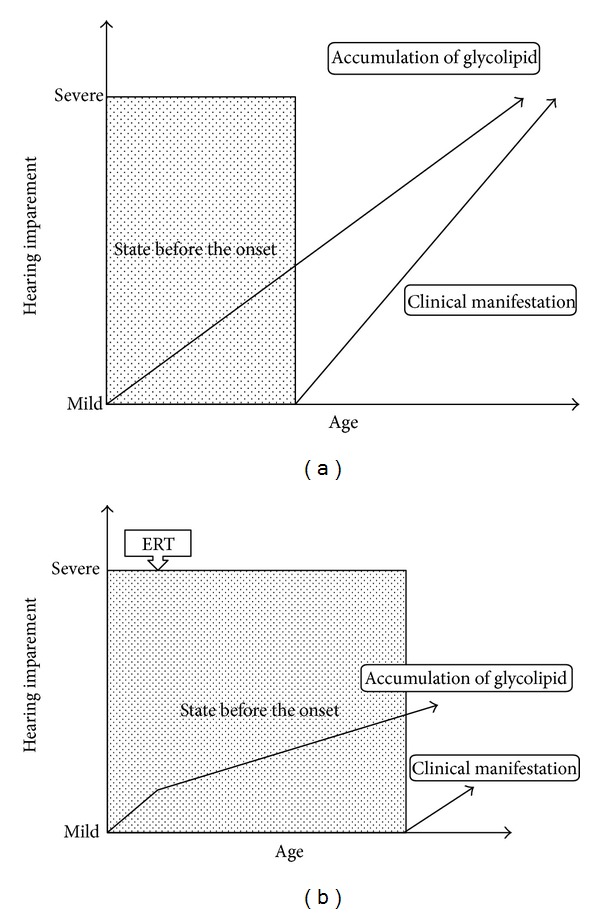
Hypothesis of the effect of ERT. (a) Natural history of Fabry disease. (b) Effect of ERT with Fabry disease.

 Because the model of Fabry disease showed a densely-stained material accumulation in the inner ear stria vascularis cell [9] and organ damage is reported to be improved by ERT [6, 7], it is reasonable for otorhinolaryngologists to expect that ERT may prevent hearing loss. Hearing loss is a symptom that markedly impairs patients' quality of life (QOL), and therefore the prevention of hearing loss is expected to contribute to the maintenance of QOL in patients with Fabry disease. On the basis of the pathologic features of Fabry disease, we hypothesized that the decrease in the accumulation of glycolipids brought about by ERT would inhibit, or delay, the manifestation of symptoms (Figure 3). Further, the progression of Fabry disease is known to vary among different individuals; these differences have been attributed to factors such as the type of Fabry disease, discrepancy in enzymatic activity, and antibodies to ERT.

 Some important findings of this study were that no eventual worsening of hearing occurred while the patients received ERT and that the incidence of SSNHL was obviously higher in this population than the incidence of sudden hearing loss in the general population. None of the patients required hearing aids, with the mean hearing level of the patients being less than 30 dB even for those who showed more than 5 dB decrease in hearing acuity. The pathologic status of SSNHL in Fabry disease remains unclear; however, some type of vascular disorder is believed to occur, similar to that observed in cerebral infarction. Although the incidence of SSNHL was higher among patients who were followedup for more than 3 years, mild cases of grade 1 severity were predominant, and the cure rate was as high as 90%. These results suggest that ERT inhibits the onset of hearing loss and SSNHL and decreases the severity of the latter. However, since this study did not include a placebo control group, the results cannot be compared against patients not receiving the therapy. Therefore, the results of the therapy over a longer period of observation should be studied before reaching a conclusion as to whether ERT is truly effective.

 In our previous study, several patients had severe hearing loss [5]. These were patients who already had severe hearing loss at the beginning of ERT and had other progressive symptoms of Fabry disease [5]. In contrast, all patients in the present study had normal hearing acuity at the beginning of ERT. Taking these features into account, the following 2 points need to be considered. First, it is possible that ERT decreases the severity of hearing loss. Second, gradually progressing hearing loss and SSNHL may have different mechanisms of onset. This speculation is supported by the finding that gradually progressing hearing loss is irreversible, whereas the cure rate of SSNHL is very high, as observed in this study. In other words, SSNHL in Fabry disease is a reversible disorder.

 The results of this study indicate that patients with Fabry disease should be periodically evaluated for hearing acuity and administered prompt treatment of SSNHL, if available, in order to maintain their QOL. A new finding of this study is that repeated hearing loss was observed as the only symptom of Fabry disease, albeit in 1 case (Case 14). This is suggestive of the diversity of the clinical presentation of Fabry disease and possibly points towards the existence of the auditory subtype of this disease, in addition to the renal subtype and the cardiac subtype. Further investigations of the pathologic condition of this disease in the light of these findings are desirable.

## 6. Conclusions

No significant worsening of hearing acuity was noted until the end of ERT. 

The incidence of SSNHL tended to be higher in patients who were followed up for a longer duration and who had renal dysfunction. SSNHL that occurred in patients on ERT for Fabry disease was relatively mild, and the cure rate for steroid therapy was 90%. The results suggest that ERT delays the onset of hearing loss and reduces the severity of SSNHL. Periodic evaluation of hearing acuity and prompt treatment of SSNHL, if any, are desirable to maintain patients' QOL.

## Figures and Tables

**Table 1:** Grades of severity of hearing loss. In Japan, the severity of SSNHL has been classified as follows.

Grade 1	Less than 40 dB
Grade 2	More than 40 dB, less than 60 dB
Grade 3	More than 60 dB, less than 90 dB
Grade 4	90 dB or more

**Table 2:** Criteria for improvement. In Japan, outcome of therapy has been classified as follows.

(1) Healing	(1) When hearing level at 0.25, 0.5, 1, 2, 4 kHz returned to less than 20 dB. (2) When hearing level returned to the same as that in the other side.
(2) Marked recovery	When the value of the arithmetic means of the hearing levels at the five above-mentioned frequencies was greater than 30 dB.
(3) Recovery	When the value of the arithmetic means of the hearing levels at the five above-mentioned frequencies was less than 30 dB and greater than 10 dB.
(4) Unchangeable	When the value of the arithmetic means of the hearing levels at the five above-mentioned frequencies was less than 10 dB. (Including exacerbation)

**Table 3:** Results along a time series. Thirteen of the 17 patients had tinnitus, while 9 had vertigo. SSNHL was found in 10 ears of 6 patients. Repetitive SSNHL was found in 3 of the 6 patients with SSNHL. The incidence of SSNHL tended to increase over the follow-up period, and all patients with renal dysfunction had SSNHL.

ID	Sex	Age	Fabry type	Tinnitus	Vertigo	SSNHL	Time to onset	SSNHL outcome	Hearing outcome	Hearing change	Renal function	Heart function	Period	Before ERT (dB)	After ERT (dB)
1	F	40	hetero	−	−	—	—	—	—	—	—	—	8 M	8.0	6.0
						—	—	—	—	—				6.0	5.0
2	F	52	hetero	+	−	—	—	—	—	—	—	—	12 M	8.0	10.0
						—	—	—	—	—				10.0	12.0
3	F	31	hetero	+	−	—	—	—	—	—	—	—	12 M	14.0	17.0
						—	—	—	—	—				14.0	15.0
4	M	55	heart	+	+	—	—	—	—	—	—	—	17 M	8.0	11.0
						—	—	—	Worth	Slow				6.0	13.0
5	M	14	classical	−	−	—	—	—	—	—	—	—	26 M	6.0	9.0
						—	—	—	—	—				6.0	9.0
6	M	38	classical	+	−	—	—	—	Worth	Slow	—	—	27 M	6.0	24.0
						—	—	—	Worth	Slow				9.0	15.0
7	F	23	hetero	+	+	—	—	—	—	—	—	—	28 M	8.0	11.0
						—	—	—	—	—				11.0	10.0
8	F	30	hetero	+	+	—	—	—	—	—	—	—	36 M	5.0	10.0
						—	—	—	—	—				5.0	9.0
9	M	25	classical	−	+	—	—	—	—	—	—	Worth	43 M	9.0	8.0
						—	—	—	—	—				11.0	8.0
10	F	47	hetero	+	+	Grade 1	54 M	Healing	—	Sudden	—	Worth	58 M	10.0	11.0
						Grade 1	41 M	Healing	—	Sudden				10.0	9.0
11	F	41	hetero	+	+	—	—	—	Worth	Sudden	—	—	67 M	12.0	18.0
						—	—	—	—	—				12.0	15.0
12	F	34	hetero	+	−	—	—	—	—	—	—	—	67 M	4.0	9.0
						Grade 1	64 M	Healing	—	Sudden				4.0	8.0
13	F	49	hetero	+	+	Grade 1/Grade 3	20/67 M	Healing/Healing	Worth	Sudden	Worth	Worth	68 M	10.0	21.0
						—	—	—	—	—				14.0	14.0
14	M	35	classical	+	−	Grade 1	48 M	Healing	—	Sudden	—	—	68 M	6.0	9.0
						Grade 1	60 M	Healing	—	Sudden				6.0	11.0
15	M	30	classical	+	+	Grade 1	66 M	Healing	—	Sudden	Worth	Worth	77 M	8.0	9.0
						—	—	—	—	—				9.0	11.0
16	M	39	classical	+	+	—	—	—	Worth	Slow	Worth	—	88 M	8.0	15.0
						Grade 1	84 M	No change	Worth	Sudden				7.0	21.0
17	M	12	classical	−	−	—	—	—	—	—	—	—	90 M	2.0	3.0
						Grade 1	90 M	Healing	Stable	Sudden				5.0	5.0
